# Analysis of CFRP Joints by Means of T-Pull Mechanical Test and Ultrasonic Defects Detection

**DOI:** 10.3390/ma11040620

**Published:** 2018-04-18

**Authors:** Caterina Casavola, Fania Palano, Francesco De Cillis, Angelo Tati, Roberto Terzi, Vincenza Luprano

**Affiliations:** 1 Politecnico di Bari, Dipartimento di Meccanica, Matematica e Management, Viale Japigia 182, 70126 Bari, Italy; francesco.decillis@poliba.it; 2 ENEA C.R. Brindisi-SS 7 km 706, 72100 Brindisi, Italy; fania.palano@enea.it (F.P.); roberto.terzi@enea.it (R.T.); vincenza.luprano@enea.it (V.L.); 3 ENEA C.R. Casaccia, Via Anguillarese 301, 00123 Rome, Italy; angelo.tati@enea.it

**Keywords:** carbon fibers reinforced polymers (CFRP), non-destructive testing (NDT), mechanical properties, ultrasonic phased array, T-joints, pull test

## Abstract

Defects detection within a composite component, with the aim of understanding and predicting its mechanical behavior, is of great importance in the aeronautical field because the irregularities of the composite material could compromise functionality. The aim of this paper is to detect defects by means of non-destructive testing (NDT) on T-pull samples made by carbon fiber reinforced polymers (CFRP) and to evaluate their effect on the mechanical response of the material. Samples, obtained from an industrial stringer having an inclined web and realized with a polymeric filler between cap and web, were subjected to ultrasonic monitoring and then to T-pull mechanical tests. All samples were tested with the same load mode and the same test configuration. An experimental set-up consisting of a semiautomatic C-scan ultrasonic mapping system with a phased array probe was designed and developed, optimizing control parameters and implementing image processing software. The present work is carried out on real composites parts that are characterized by having their intrinsic defectiveness, as opposed to the previous similar results in the literature mainly obtained on composite parts with artificially produced defects. In fact, although samples under study were realized free from defects, ultrasonic mapping found defectiveness inside the material. Moreover, the ultrasonic inspection could be useful in detecting both the location and size of defects. Experimental data were critically analyzed and qualitatively correlated with results of T-pull mechanical tests in order to better understand and explain mechanical behavior in terms of fracture mode.

## 1. Introduction

Composite materials represent a high-performance material for engineering applications, widely used in the aeronautical field. They are expected to guarantee better performances and reduced weight compared to conventional metallic alloys [1,2,3]. Nevertheless, the mechanical characteristics of composites depend not only on the matrix and fibers properties, but also on the combination of them and on different other variables related also to the manufacturing process and to the final quality of parts. The most important benefit respect to metal alloys is connected to the possibility of designing them ad hoc for the specific application that they are intended. On the other hand, the heaviest disadvantage to take into account is related to the high level of intrinsic defects inside the material. For these reasons, the use of composite in aeronautical application has indicated until now a high level of possibilities in design due to the almost unlimited combinations of composite materials and fiber patterns, which suggests the importance of both an extensive campaign of experimental tests (static load, cycle fatigue, strain rate and temperatures, moisture conditions, etc.) and non-destructive inspection to mechanically characterize materials [4]. Consequently, experimental tests continue to play a significant role in the qualification process of composite materials by using both traditional [5] and hybrid techniques [6]. 

The importance of a non-destructive evaluation (NDE) of composites and their structures grows with the increasing use of composite materials and it is necessary to ensure industry requirements for safety and reliability of materials and components.

During the production phase and in service for critical structures, it is essential to use non-destructive tests to assess the quality and the health of the product. The non-destructive tests do not measure strength directly but measures a parameter that can be correlated to strength. It is, therefore, essential that a suitable NDT method is chosen and that its results are correctly interpreted. The acoustic imaging method is one of the more disseminated choices among non-destructive techniques because it allows not only to detect the presence of defects but also to characterize them in terms of size, shape, and location [7].

The propagation characteristics of ultrasonic waves are used to determine material properties, to detect and characterize the surface and subsurface flaws. They are also suitable for quality control and for the estimation of the adhesion level between composite layers.

In the pulse–echo method an ultrasonic pulsed wave is generated by a probe and propagates into a specimen with the ultrasonic velocity corresponding to the material concerned. Part of the ultrasound will be reflected if it strikes an obstacle in the form of an inhomogeneity and, if this is not too large, the remainder will travel further to a boundary of the specimen and will be reflected to a receiver. The signal obtained from the receiver is displayed as a peak on a base line. The horizontal sweep is proportional to the time, so that the transit time of the pulse to and from the reflector and to and from the back wall, correspond respectively to the distances on the screen from the initial peak to the echo peaks corresponding to the reflector and back wall.

The ultrasonic technique is the most applied method in monitoring the integrity and in the identification of damage in composite aeronautical structures [8,9,10,11,12]. Moreover, it is of fundamental importance in the design of composite aeronautical structures, to predict, by means of NDTs, the reduction of the mechanical strength of the material in the presence of defects, above all in terms of ultrasonic signal attenuation [13,14,15]. The phased array ultrasonic technique allows to detect all the typical defects of the composite materials and to determine their size and position with high accuracy [11,12]. It is also faster and easier in respect to the traditional pulse–echo method. A phased array system utilizes the wave physics principle of phasing, varying the time between a series of outgoing ultrasonic waves. The probe elements will be pulsed in groups to improve effective sensitivity by increasing aperture, which reduces unwanted beam spreading and enables sharper focusing. The C-scan is a 2D image representation displaying an ultrasonic wave signal acquired point-by-point from the structure and it can be used to map the component area. For automated inspection, the C-scan system consists of motorized scanner to move an ultrasonic probe over the structure. Other NDT techniques, such as thermography, can be applied to detect defect distribution in carbon fiber reinforced polymers (CFRP) materials, but ultrasonic testing (UT) methods seems to offer better results and detect deeper defects [16]. In this paper, a preliminary study to critically correlate the mechanical behavior of CFRP specimens with a real defects map detected by ultrasonic inspection has been carried out. As few papers focus on this comparative analysis, the aim of this paper is to shed light on the correlation between mechanical response and intrinsic defectiveness, which seems to be interesting in view of improving the performance required to composite T-joints in the aerospace industry. 

The correlation between defect maps detected by ultrasonic analysis and mechanical results has been qualitatively carried on. The presence, the size, and particularly the location of defects were correlated with the fracture mode, meaning the side on which the rupture occurred and the way in which it propagated.

The geometry of tested specimens is a T-joint, which is a widely used composite joint in aeronautical structural applications. They are made of three parts (the skin, the web, and the cap) and represent a very critical connection for the difficulties in manufacturing good quality parts (that is without defects) and consequently for their mechanical strength both static and fatigue. The high level of attention on these T-joints is also due to the absence of specific test standards, which complicates the procedure of experimentally detecting the mechanical response. For this reason, the load system was created ad hoc to realize both the constraint and load conditions required by the project specifications. 

Some detailed information about materials and the manufacturing process have to be hidden due to a non-disclosure agreement signed with the aeronautical industrial partner involved in the research project. The work has been organized as follows: nine samples, considered free from defect, under study made by the CFRP composite were first subjected to ultrasonic control and subsequently to T-pull mechanical tests and correlate the ultrasonic data with the mechanical response of the component. In particular, crack area and fracture behavior were analyzed. The non-destructive ultrasonic inspection required a preliminary design and realization of an experimental set up to perform non-destructive tests using a Phased Array Technique on the CFRP composite specimens. The experimental set-up consists of a semiautomatic ultrasonic C-scan mapping system with a phased array probe realized ad hoc. Also, the control parameters were optimized and image analysis processing software was used.

## 2. Materials and Methods

### 2.1. Experimental Plan

Tests were conducted on nine T-shaped specimens taken from an industrial stringer assy made of co-cured CRFP composite material that were supposedly sound in order to study their mechanical strength in T-Pull tests.

**Figure 1:**
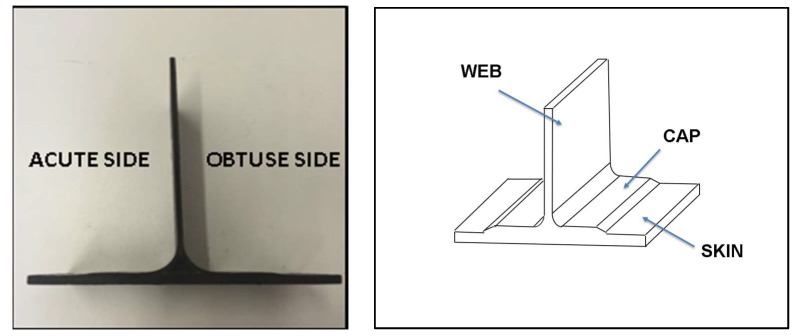
T-Pull specimen and technical nomenclature.

Geometry and nomenclature of T-shaped specimen studied in this work are shown in Figure 1.

Layups of specimens are shown below:

- Skin: [45/-45/0/45/-45/90/45/-45/0/-45/45/90] s (24 ply)
- Spar web: [45/-45/0/90/0/45/-45/0] s (16 ply)
- Spar cap: [45/-45/0/90/0/45/-45/0] (8 ply)
- Single ply thickness: 0.186 mm

The dimensions of the specimens (height, length and width of skin and cap, thickness) are listed in Table 1.

All tested specimens have been cut from a stringer component with a 5° sloping web with respect to the skin and polymeric filler between cap and web. They are characterized by an acute side and obtuse one (Figure 1).

A preliminary visual inspection revealed that almost all of the samples have some defects in the area of cutting that potentially could be responsible of cracks nucleation and delamination under mechanical stresses. 

### 2.2. Ultrasonic Testing Setup

Using ultrasonic ND controls, the presence of delaminations and porosity in CFRP T-shaped components was investigated. 

CFRP T-joints were characterized through ultrasonic imaging (UT) analysis in order to assess the defects map before being subjected to mechanical tests.

The mapping of each sample using non-destructive testing has been obtained. Then, the fracture area visual images that were obtained after the mechanical tests have been compared with defects highlighted with non-destructive controls. 

**Figure 2:**
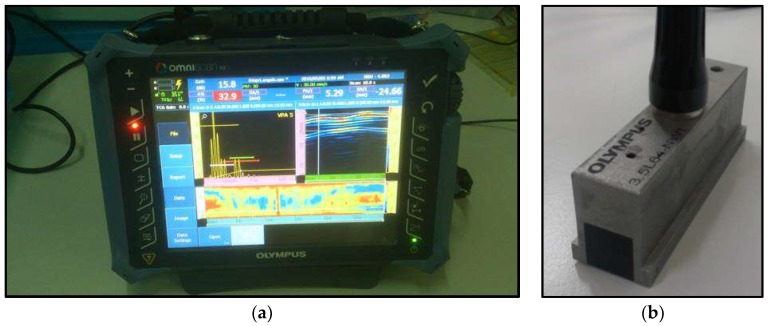
(**a**) Omniscan MX2 pulser receiver; (**b**) 3.5 MHz phased array probe.

The experimental set-up is based on the Olympus OmniScan MX2 system (Figure 2a) with a Phased Array module able to guarantee high performances in automatic applications.

The samples were inspected by means of a phased array flat probe, operating at a frequency of 3.5 MHz (Figure 2b). A phased array probe consists of a transducer assembly with 64 small individual elements that can each be pulsed separately, but are divided in groups of eight. It is arranged in a linear array and is designed for immersion use with sound coupling through a water path. Software, known as a focal law calculator, establishes specific delay times for firing each group of elements in order to generate the desired beam shape, taking into account probe and wedge characteristics as well as the geometry and acoustical properties of the tested material. The programmed pulsing sequence selected by the instrument’s operating software then launches a number of individual wave fronts in the test material. These wave fronts in turn combine constructively and destructively into a single primary wave front that travels through the test material and reflects off cracks, discontinuities, back walls, and other material boundaries like any conventional ultrasonic wave. 

The C-scan, a two-dimensional presentation of data displayed as a top or planar view of a test piece where color represents the gated signal amplitude at each point in the test piece, maps its x-y position. With conventional instruments, the single-element transducer must be moved in an x-y raster scan pattern over the test piece. In the phased array system, the probe is moved physically along one axis while the beam electronically scans along the other. 

**Figure 3:**
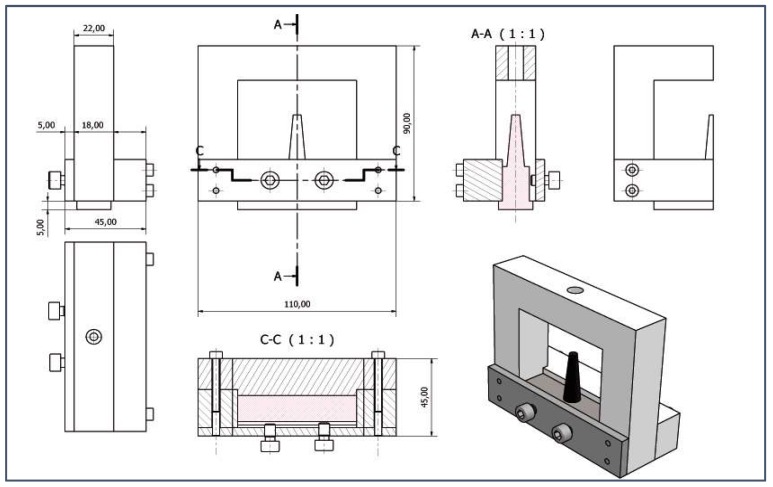
Homemade designed Probe-Holder.

A homemade probe-holder (Figure 3), mounted on the movement axis, was designed and realized in order to use the automatic control of the probe.

The scan operation was done by using a Mini-Wheel encoder, which is connected to the pulse-receiver instrument, to synchronize the probe data acquisition with the probe unidirectional motion. The scans are displayed on C-scan diagrams, which present a two-dimensional view of data displayed in a top or planar view on a specimen, monitoring the data in the XY position. 

**Figure 4:**
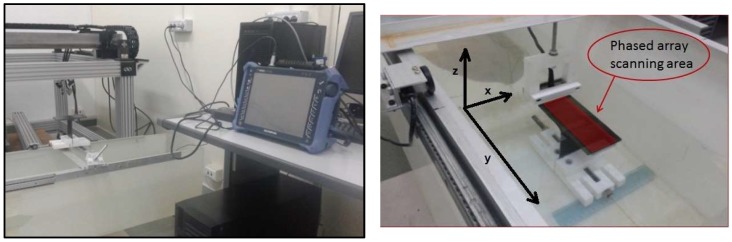
Semiautomatic C-Scan Setup.

A semiautomatic system (Figure 4) with one axis encoder guide mounted on a large Plexiglas tank (110 × 40 × 35 cm) was designed and developed. The guide allows a probe position on axes x, y and z, by means of suitable sliding and fixing systems. The encoder was placed on the y axis.

The CRFP T-shaped specimen is analyzed by the probe placed on a Teflon made support. Both specimen and probe are immersed in water. The support, containing the probe, slides on the specimen surface using the water as a coupling fluid.

Particular attention was paid to the positioning of the samples with respect to the probe, due to the inclination of the web with respect to the skin.

The Omniscan instrumentation is equipped with an encoder input for the synchronization of the probe displacement signal. Once the encoder has been calibrated, the system is able to automatically acquire the ultrasonic signals during the axis movement.

The probe sensitivity and the ultrasonic velocity (3010 m/s) in the material under study has been calibrated before performing the ultrasonic testing.

Moreover, in order to visualize the reflector real dimension independently from its position, the TCG (Time Corrected Gain) reference curve has been built, based on same size reflector response as a function of distance. The same gain value (8 dB) has been set for all of the tested specimens.

### 2.3. Mechanical Testing Setup

**Figure 5:**
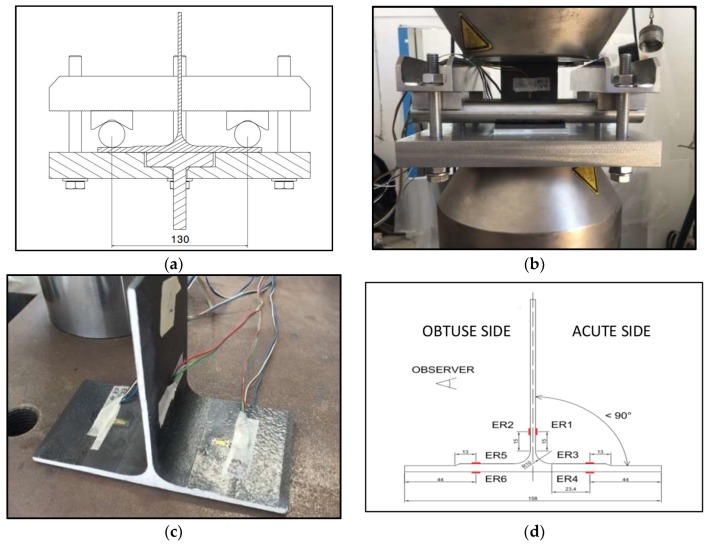
(**a**) Geometry and (**b**) image of loading system; (**c**) T-Pull specimen and (**d**) strain gage locations.

Experimental tests were executed using the loading system for T-Pull tests shown in Figure 5a.

Since there is no standard setup for a pull-test of CRFP T-joints, an ad hoc load system has been designed and implemented.

In the absence of a specific standard for this type of tests, the loading system has been designed and realized ad-hoc in order to study the tensile strength of the web. It consists of a support base on which the skin of the T-shaped component is placed and constrained in its position by two 25 mm diameter steel rollers; these should guarantee a linear contact with the surface where the layer of the web overlap with those of the cap.

Figure 5a shows the positioning of the specimen in the loading system, where the interaxis between the two blocking rollers is 130 mm. Figure 5b shows the complete set up: the load system installed in the testing machine includes the specimen. In order to highlight and monitor the trigger of the delamination, the specimens were painted with white paint on the cross-sections. A video recording of each test was carried out, always filming the white-painted side of the specimen. In this way it was possible to isolate images relating to the beginning of the delamination. Uniaxial electrical strain gages (ERi) for composite materials (350 Ohm, 6 mm measuring grid) were bonded in the area that is expected to be more critical (Figure 5c,d). 

Strain gage location is as follow: ER1 and ER2, ER3 and ER5, ER4, and ER6 are symmetrically located with respect to web; ER3 and ER4 are located respectively on the top and bottom of the cap. ER5 and ER6 are the same.

It has been observed that the inclination of the web with respect to the skin leads to the occurrence of bending deformations when it is clamped in the testing machine. These considerations led us to choose a configuration with six strain gauges. The ER1 strain gauge always lies on the side of the web that forms an angle less than 90° from the skin (acute side). Once the loading system has been installed in the testing machine, the observer positioned at the front sees the side with ER2.

The tests were conducted at a speed of 1 mm/min and a sampling frequency of 10 Hz. The test apparatus used is a Schenck two-column servohydraulic machine (Schenck, Darmstad, Germany) equipped with a 250 kN load cell, hydraulic clamping up to 300 bar, MTS Flex 40 controller, and System 5000 strain gauges data acquisition. 

## 3. Results

Experimental results of T-pull mechanical tests are summarized in Table 2 where maximum delamination load values and the corresponding displacement are indicated. 

**Figure 6:**
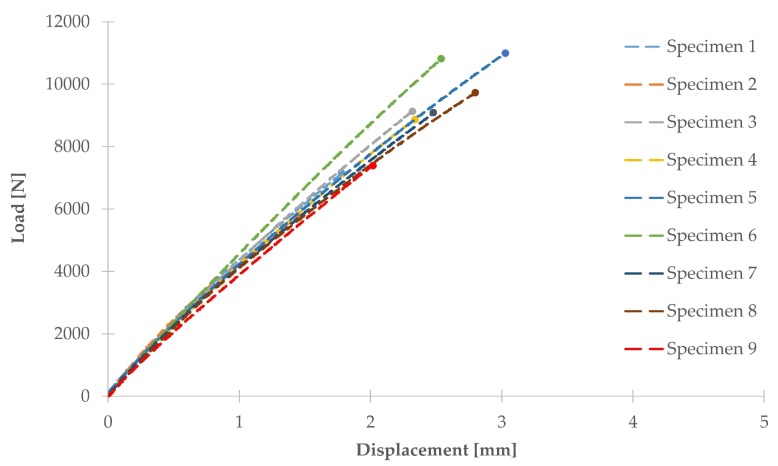
Load versus displacement curves.

**Figure 7:**
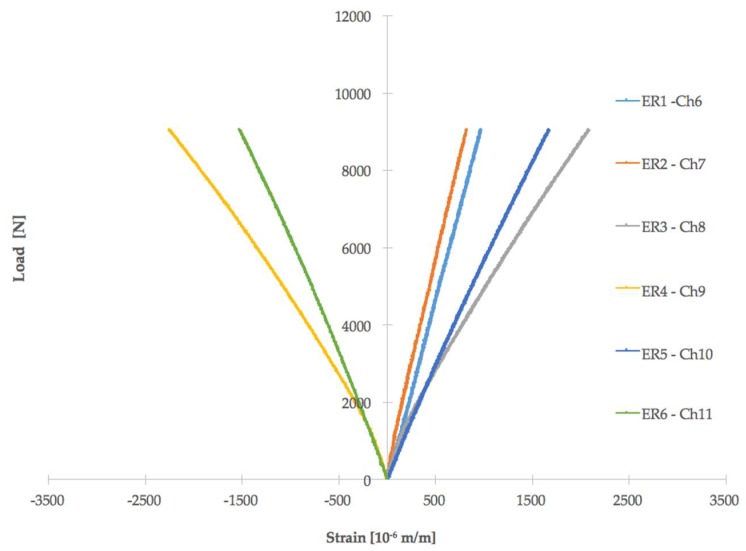
Strain gages value recorded during mechanical tests for specimen number 3.

Nine T-pull specimens were analyzed. Load versus displacement curves for all of the tested specimens are shown in Figure 6. As an example, Figure 7 describes strain gages values recorded during mechanical tests for one specimen (specimen number 3). ER1 and ER2 (located on the web) reveal a lower level of strain with respect to those on the skin and each couple of strain gages on the skin are symmetrically located with respect to web (that is ER3 with respect to ER5 and ER4 with respect to ER6) and confirm that the acute side is more deformed (this can be observed for all specimens). Moreover, it can be observed that at a lower mechanical load, i.e., less than 3000 N, the strain gages values are quite symmetric. At a higher load, close to the final fracture, as usual, the material strength changes and strain gage values of ER3, ER4, ER5, and ER6 diverge. In view of this consideration, Table 1 reports also the location (acute or obtuse side) of the final delamination. Specimens 2, 7, and 9 delaminated on the obtuse side and this is a quite unexpected behavior that could be explained on the basis of ultrasonic data more than strain gage findings.

Ultrasonic and T-pull test results are detailed in the next paragraphs. The samples are divided in two categories: specimens in which a fracture has occurred on the acute side and those in which it has arisen on the obtuse one.

**Figure 8:**
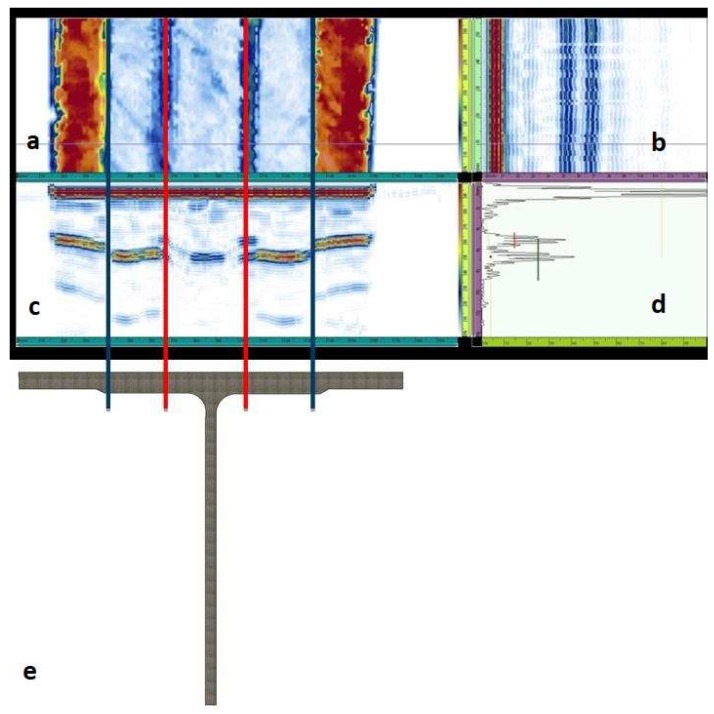
Correspondence between ultrasonic images and specimen typical geometry: (**a**) C-scan; (**b**) D-scan; (**c**) B-scan; (**d**) A-scan; and (**e**) specimen geometry. Red lines localize the defects position and blue lines correspond to the thickness variation at the skin-cap interface.

**Figure 9:**
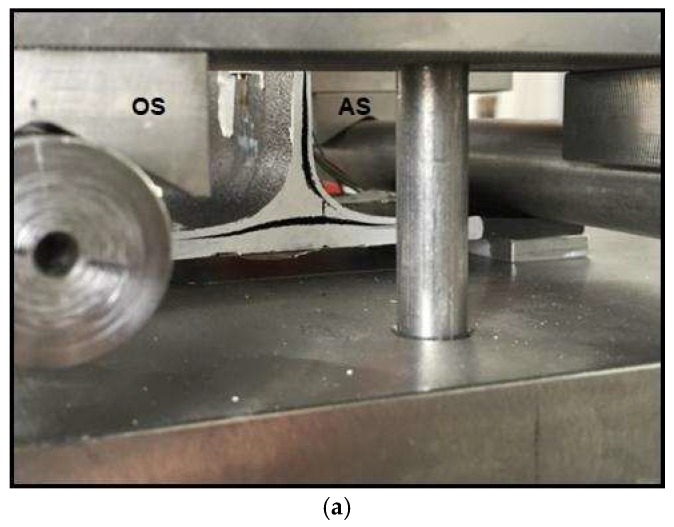

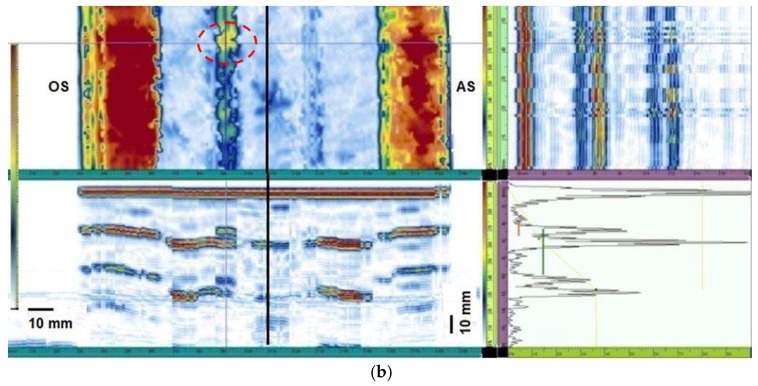
(**a**) Specimen 3 after delamination fracture; (**b**) ultrasonic testing (UT) images before the breaking; the red circle shows an irregular defected area.

**Figure 10:**
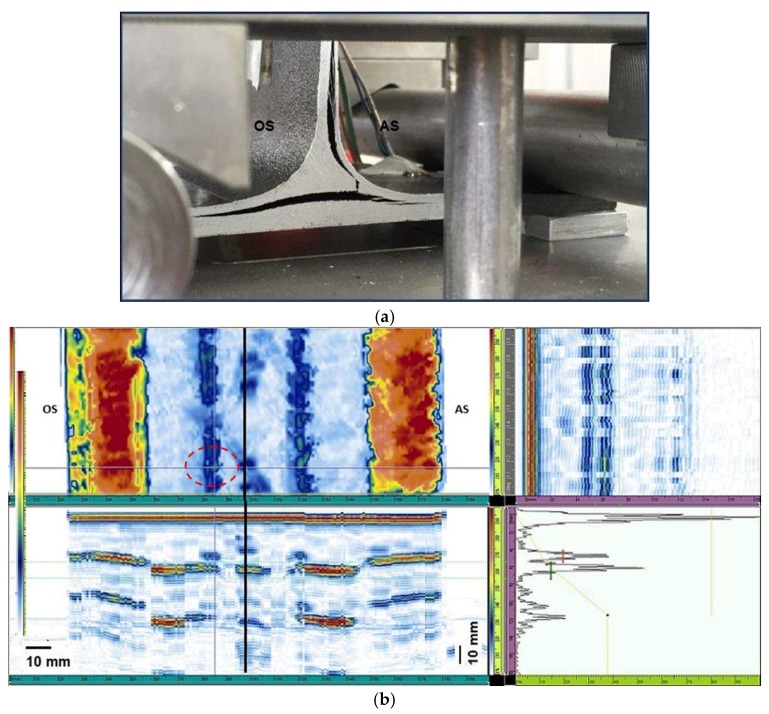
(**a**) Specimen 4 after delamination fracture; (**b**) UT images before the breaking; the red circle shows an irregular defected area.

**Figure 11:**
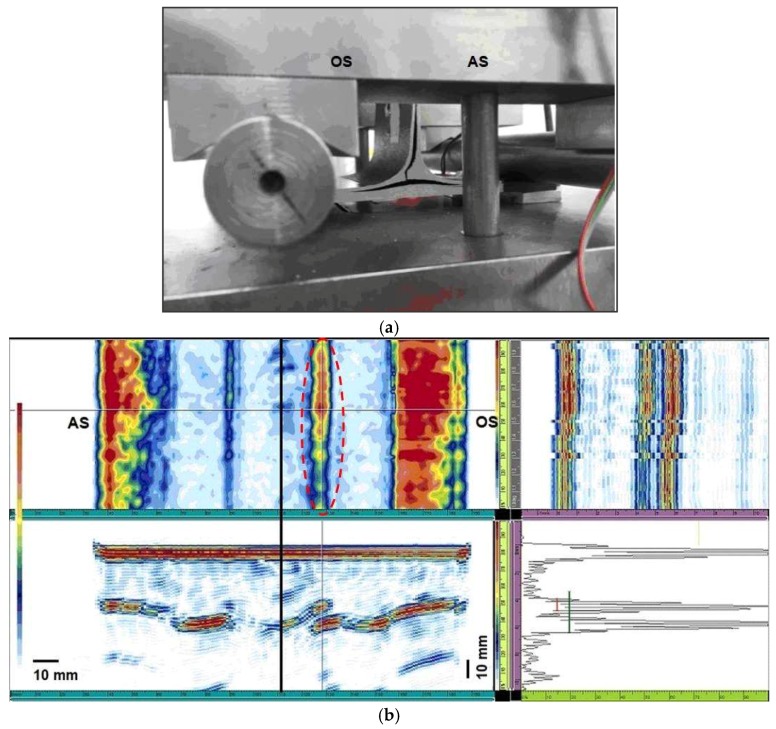
(**a**) Specimen 9 after delamination fracture; (**b**) UT images before the breaking; the red circle shows the continuous defected area.

**Figure 12:**
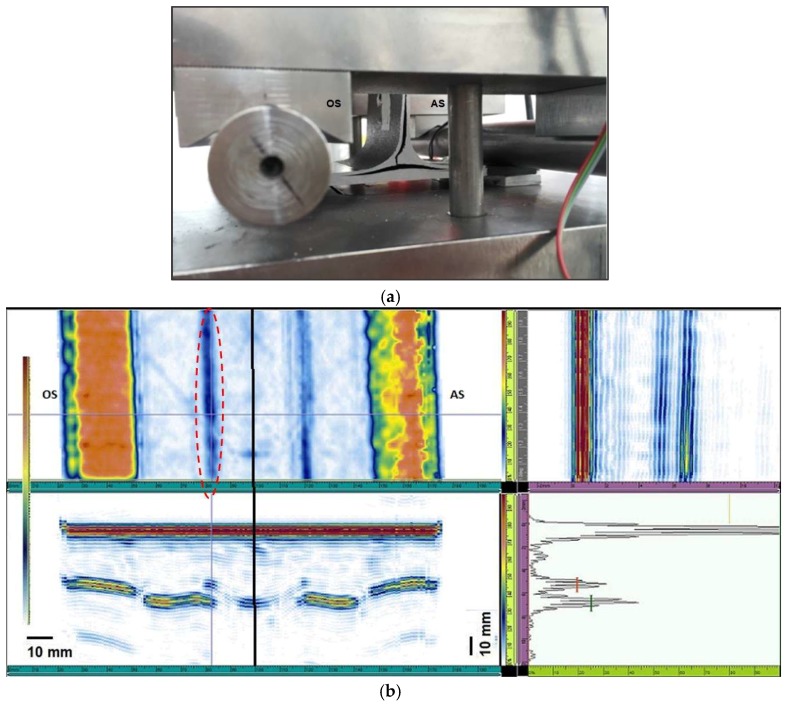
(**a**) Specimen 7 after delamination fracture; (**b**) UT images before the breaking; the red circle shows the continuous defected area.

Ultrasonic data have been acquired, saved, and processed with the Omniscan MX2 flaw detector. Graphic representations of the component inner part in ecographic form are shown in Figure 8, Figure 9, Figure 10, Figure 11 and Figure 12. OmniPC software allows for the offline elaboration of the .opd files saved by Omniscan MX2.

All UT images are obtained selecting the distance of the slice from the probe, calculated by the software using the wave propagation speed and the time of flight, corresponding to the specimen second interface. C-scan images of the section of interest show, according to the scale, areas with a great amplitude of the reflected signal (pulse height in the A-scan), and areas with low amplitude of the signal. The first type of images (yellow-red color bar) reveals areas in which the defectiveness has occurred, the second type refers to an area with different material (Figure 8, Figure 9, Figure 10, Figure 11 and Figure 12). 

An important parameter is the extension of the defect along the specimen width. In few cases, in fact, the defectiveness intersects the whole width of the specimen even if the corresponding signal has low amplitude (i.e., Figure 11).

The images allow us to appreciate significant defects in all of the investigated samples. 

C-scan nondestructive analysis, realized before mechanical tests by means of the phased array ultrasonic technique, reveals a discontinuity in correspondence to the T-joint (Figure 8a,c,e) in all the specimens. For some specimens this irregularity extends to the whole sample width and it is characterized by high peak amplitude in several points at the filler/cap interface. 

In all the images produced from the ultrasonic test, C-scan is displayed on the top-left, D-scan (B-scan along a longitudinal plane) on top-right, while B-scan is shown on bottom-left and A-scan on the bottom-right (Figure 8a–d). Gate I is placed on the water/material interface boundary echo, gate B is on back wall echo and gate A in the component thickness, moving it according to the requirements in order to display the examined detail on the C-scan. The higher peak amplitude in the A-Scan signal is displayed in C-scan images by a yellow-red color palette, whereas attenuated signals appear as a green-blue color palette. Tilted surfaces compared to the beam axis direction produce an increase in attenuation. Therefore, the boundary echo in correspondence to the T section and the fillet radii in correspondence to the thickness variation are not visible. 

The more representative ultrasonic image can be considered the C-scan. On this representation, the section displayed in the B-scan corresponds to the horizontal grey line on the C-scan, whereas the section represented in the D-scan corresponds to the vertical grey line on the C-scan (Figure 8a–c).

Moreover, the defect point corresponding to the maximum ultrasonic signal amplitude has been chosen (perpendicular grey lines intersection point) and the related A-scan has been displayed on the bottom-right (i.e., Figure 8d).

Figure 8, as an example, clarifies the correspondence between the detected defects zones by ultrasound images and the scanned sections of the T-joints real component (Figure 8e). The red lines in Figure 8a clearly localize the defects position that occur in the interface zone skin-web-filler. This area is a critical one (in fact, mechanical fractures started always from here) and the information about the position of defects and their dimensions are useful to reveal imperfections related to the manufacturing process that cause delamination in the specimen when subjected to mechanical load. The extension of this area can be qualitatively correlated to the mechanical strength of the specimen. The blue lines correspond to the thickness variation at the skin-cap interface and represent a change in the geometry more than a defect (in fact, no mechanical fractures started from this zone).

The greater stress concentration factor due to a geometric configuration on the acute side should led to a fracture on this side. But the presence of significant defects on the obtuse side and their extension to the whole specimen width influenced the fracture mode and led in the case of specimens 2, 7, and 9 to a rupture on the obtuse side.

So, in this the specimens have been divided in two categories: the failed ones on the acute side, in line with expectations, and the broken ones on the obtuse side. 

### 3.1. Acute Side Fracture

The specimens 1, 3, 4, 5, 6, and 8 submitted to T-pull tests show similar fracture modes (Figure 9a and Figure 10a).

For all these samples, delamination initiation has occurred on the web side that forms an acute angle with the skin. Delamination propagation has proceeded both along the vertical direction that along the skin plies in the horizontal direction.

Specimens 1, 3, 6, and 8 show also a similar acoustic behavior. The ultrasonic images related to sample 3 are displayed as an example (Figure 9).

The presence of a defect on the obtuse side was detected. The defect on the obtuse side is irregular and not continuous and the area corresponding to the higher ultrasonic signal (red-yellow palette) is indicated by a red circle. The vertical axis indicated the web position is displayed by a thick black line. Also, in the other analyzed specimens the defect appears not continuous and with small and variable dimensions. In the specimens 1, 3, 6, and 8 the ultrasonic signal amplitude on the defect reaches 50% of the maximum amplitude. 

For these samples, the breaking occurred on the acute side. In fact, slight and discontinuous defectiveness has been observed on the obtuse side, which has not produced neither significant stress concentration on the obtuse side nor a relevant bonding weakening. The high fracture stress for the specimens 3, 6 and 8 (Table 1) seems to confirm that the observed defectiveness has not significantly compromised the bonding strength.

In the sample 1, it is also possible to identify a central flaw having 65% peak amplitude and a continuous defect on acute side. In this case, the low breaking load could be due to the identified defectiveness, both in the middle and on the two sides of the sample.

For specimen 4, the ultrasonic images (Figure 10b, C-scan) show the presence of a slight imperfection on both sides: the defects appear not continuous and characterized by variable dimensions.

The ultrasonic beam amplitude corresponding to the defect position on the obtuse side (Figure 10b, C-scan right side) reaches the maximum, about 38%, in the displayed A-Scan (Figure 10b).

An irregular defectiveness area was detected on the acute side (Figure 10b, C-scan left side), that could have influenced specimen breaking on the acute side at a low failure stress value (Table 1).

For sample 5, the ultrasonic images reveal the presence of a defected area on the obtuse side. The defect seems not continuous and characterized by irregular dimensions.

Ultrasonic signal amplitude corresponding to the obtuse side defect is about 32% and its dimension is the same along the whole sample width. It is also possible to detect a central discontinuity.

The breaking occurred on the acute side and appears coherent with the ultrasonic results. In fact, an irregular discontinuity was observed on the obtuse side, which has not produced neither a significant stress concentration nor an important bonding weakening. 

### 3.2. Obtuse Side Fracture

The specimens 2, 7, and 9 present a similar fracture mode. For all the samples, the delamination initiation has occurred at the web side that forms an angle >90° with the skin. Delamination propagation continues both along the vertical direction and the skin plies horizontal direction (Figure 11a and Figure 12a). Specimens 2 and 9 show, also, a similar acoustic behavior. UT images of sample 9 are reported in Figure 11.

A continuous and large defect was detected on sample 2 and 9 on the obtuse side (Figure 11b, C-scan right side). The dimensions of these defects (Figure 11b, A-scan) are constant along the whole specimen width and their peak amplitude is more than 50% and 80% all over the whole defect, respectively for specimens 2 and 9. Furthermore, in specimen 9 it is possible to recognize a central discontinuity in correspondence of the web axis (Figure 11b, C-scan) that overlaps the other flaws identified on the obtuse side.

The ultrasonic images (Figure 12b) reveal in sample 7 the presence of a regular and continuous defectiveness on both sides, but slightly more significant on the obtuse side (Figure 11b, C-scan left side): the defect appears regular and continuous. Moreover, it is possible to identify a central discontinuity (Figure 11b, C-scan).

The sample breaking that occurred on the obtuse side seems to be coherent with the relevant defectiveness (on the obtuse side).

The breaking of specimens 2, 7, and 9, arisen on the obtuse side, seems to be coherent with the continuous and regular defectiveness identified on the obtuse side, which compromised the bonding mechanical strength.

### 3.3. Remarks on Experimental Mechanical Test and Ultrasonic Images 

Ultrasonic images show in some cases the presence of regular and continuous defects located on the obtuse side of specimens 2, 7, and 9. Continuous defects are also slightly evident on the acute side of specimens 2 and 7, but they appear less relevant. In fact, in the case of specimens 2, 7, and 9, the extension of defects to the whole specimen width influenced the fracture mode and led to a rupture on the obtuse side, also if the greater stress concentration factor due to geometric configuration on acute side could have led to a fracture on this side. 

Furthermore, it can be observed that continuous defects on the acute side are quite evident in the case of specimens 1 and 4.

These observations are collected in Table 3. 

In order to clarify the intensity of the defectiveness on both the acute and obtuse sides of the specimen, the ultrasonic images have been elaborated starting from the C-scan map of each specimen and added in Table 3. These images present palette ranges between 0–35 in percentage with respect to the CAP background echo to highlight the different types of defects (linear or jagged delamination).

Summarizing, the main finding of the present work is in the relation between the typology of defects (continuous vs. discontinuous) and the location of the final fracture of the specimens. A discontinuous defectiveness along the obtuse side does not seem to rule out the mechanical response of specimens, in fact all other specimens (that is specimens 1, 3, 4, 5, 6, 8) delaminate on the acute side, as expected. On the contrary, continuous defectivity along the obtuse side establishes the fracture face.

Similarly, it should be noted that continuous defects on the acute side, as in the case of specimens 1 and 4, could play an important role in the delamination process, justifying the lower delamination load of these specimens with respect to specimens 3, 5, 6, and 8 (Table 2).

The crack propagation and fracture mode, instead, are similar for the two categories due to the defects’ localization along the filler/cap interface on both sides.

On the basis of these considerations, it seems that morphology of the defectiveness (that is a continuous or discontinuous defect) rules the mechanical behavior of the specimen. 

For the sake of completeness, it can be observed that the results of the present study on the defects of composite T-joints and their relation with mechanical strength is consistent with well-known data in the technical literature in the case of metals. In fact, defects produced in metal welded joints are considered relevant not only for their size, but also and above all for how they appear distributed and aggregated in the entire joint section [17,18,19].

## 4. Conclusions

The non-destructive C-scan control realized by means of a phased array technique has allowed us to detect the presence of defects within CFRP T-pull samples.

The specimens provided by the industrial manufacturer were considered to be free from defects, but the ultrasound mapping detected internal defects in the material as a delamination at the interface between the inner core and surrounding reinforcing fibers. The ultrasonic testing procedure allowed detecting the position and the morphology of the defect.

The presence, particularly the morphology of the defects, has been qualitatively correlated with the fracture mode mean as the side on which the final rupture has occurred and the way in which it has propagated.

The crack propagation is similar for the two categories due to the defects of localization along the filler/cap interface on both sides.

The ultrasonic results were analyzed and compared (Table 3) with the T-pull mechanical tests in terms of fracture mode, showing in most of the samples a relation between the detected morphology of defects (continuous or discontinuous) and the fracture face (delamination on the acute side or obtuse side). The experimental evidence of the mechanical test, also in analogy with metals, suggests that the morphology of the defect is relevant information.

An interesting preliminary relation between the discontinuity of the defect and the mechanical results can be supposed, even if the amount of specimens is small. 

It is worth underlying that the present study only demonstrates that the defectiveness continuity and regularity are characteristics that can mainly affect the fracture side of the specimens. 

Further experimentations exploring the possibility to use such a method to predict the fracture side and also the delamination process are still needed, mostly concerning the ultrasonic image analysis. The promising results illustrated in this study constitute a first step to developing a new procedure for quick, easy, and useful inspection of the CFRP T-joint. 

## Figures and Tables

**Table 1:** Dimensions of T-Pull specimens.

Height (mm)	Cap Length (mm)	Skin Length (mm)	Skin Width (mm)	Cap Thk (mm)	Skin Thk (mm)	Web Thk (mm)
134	96	157	76	6.1	4.7	3.2

**Table 2:** T-Pull mechanical test results.

Specimen	Delamination Load (N)	Crosshead Displacement (mm)	Breaking Side
1	7172	1.79	acute
2	5784	1.46	obtuse
3	9050	2.32	acute
4	8872	2.33	acute
5	11023	3.03	acute
6	11145	2.55	acute
7	9539	2.65	obtuse
8	9729	2.8	acute
9	6961	2.02	obtuse
Mean	8808	2.33	-
Standard deviation	1839	0.50	-

**Table 3:** Correlation between the breaking side of specimen and the defectiveness characteristics.

Specimen	Breaking Side	Defect Typology on Obtuse Side	Defect Typology on Acute Side	C-Scan Images (Threshold 35%)
**1**	**acute**	discontinuous	**continuous**	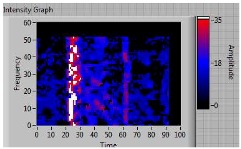 Obtuse side SX—Acute side DX
2	**obtuse**	**continuous**	continuous (but less relevant than obtuse side)	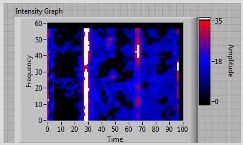 Obtuse side SX—Acute side DX
3	acute	discontinuous	discontinuous	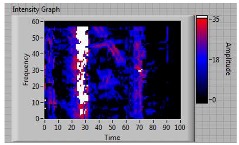 Obtuse side SX—Acute side DX
**4**	**acute**	discontinuous	**continuous**	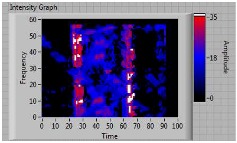 Obtuse side SX—Acute side DX
5	acute	discontinuous	discontinuous	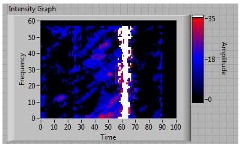 Acute side SX—Obtuse side DX
6	acute	discontinuous	discontinuous	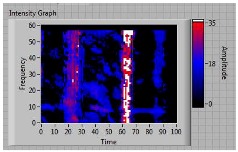 Acute side SX—Obtuse side DX
7	**obtuse**	**continuous**	Continuous (but less relevant than obtuse side)	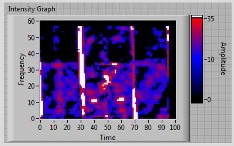 Obtuse side SX—Acute side DX
8	acute	discontinuous	discontinuous	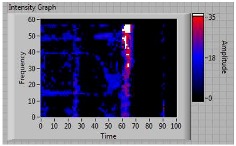 Acute side SX—Obtuse side DX
9	**obtuse**	**continuous**	discontinuous	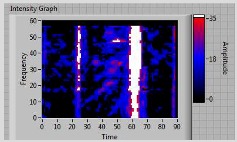 Acute side SX—Obtuse side DX

## References

[1] Peters S.T. (1998). Handbook of Composites.

[2] Cox B., Flanagan G. (1997). Handbook of Analytical Methods for Textile Composites.

[3] Cantwell W.J., Morton J. (1991). The impact resistance of composite materials—A review. Composites.

[4] Carlsson L.A., Donald F., Adams R. (2014). Byron Pipes. Experimental Characterization of Advanced Composite Materials.

[5] Barile C., Casavola C., Pappalettere C. (2017). The influence of stitching and unconventional fibres orientation on the tensile properties of CFRP laminates. Compos. Part B Eng..

[6] Barile C., Casavola C., Pappalettera G., Pappalettere C. (2013). Hybrid characterization of laminated wood with ESPI and optimization methods. Conference Proceedings of the Society for Experimental Mechanics Series.

[7] Maeva E.Y., Severina I., Wehbe H., Erlewein J. Ultrasonic Imaging Techniques to Evaluate Quality of Fiber Reinforced Composite Materials and their Adhesive Joints. Proceedings of the 5th Pan American Conference for NDT.

[8] Avinash S.H., Singh N.G., Makarand J. Damage detection methodology using ultrasonic non-destructive testing for composites structures. Proceedings of the National Seminar & Exhibition on Non-Destructive Evaluation (NDE 2011).

[9] Jolly M.R., Prabhakar A., Sturzu B., Hollstein K., Singh R., Thomas S., Foote B., Shaw A. (2015). Review of Non-destructive Testing (NDT) Techniques and their applicability to thick walled composites. Procedia CIRP.

[10] Gholizadeh S. (2016). A review of non-destructive testing methods of composite materials. Procedia Struct. Integr..

[11] Boychuk A.S., Generalov A.S., Stepanov A.V. Nondestructive testing of FRP by using phased array ultrasonic technology. Proceedings of the 12th International Conference of the Slovenian Society for Non-Destructive Testing.

[12] Scarponi C., Briotti G. (2000). Ultrasonic technique for the evaluation of delaminations on CFRP, GFRP, KFRP composite materials. Compos. Part B.

[13] Prakash R. (1981). Significance of defects in the fatigue failure of carbon fibre reinforced plastics. Fibre Sci. Technol..

[14] De Almeida S.F.M. (1994). Effect of void content on the strength of composite laminates. Compos. Struct..

[15] Grimberg R., Savin A., Steigmann R., Bruma A., Barbanescu P.D., Salavastru D.P. Determination of CFRP’s mechanichal properties using ultrasound methods. Proceedings of the 5th International Workshop NDT in Progress, Meeting of NDT Experts.

[16] Carofalo A., Dattoma V., Palano F., Panella F.W. ND testing advances on CFRP with ultrasonic and thermal techniques. Proceedings of the 16th European Conference on Composite Materials, ECCM.

[17] Casavola C., Giordano V., Pappalettere C. (2012). Mechanical behavior of as welded and smooth cord titanium welded joints. J. Struct. Integr. Life.

[18] Murakami Y. (2012). Material defects as the basis of fatigue design. Int. J. Fatigue.

[19] Hu K.X., Chandra A., Huang Y. (1993). Multiple void-crack interaction. Int. J. Solids Struct..

